# Educational intervention to prevent postoperative complications and improve functional outcomes in patients with transurethral resection of the prostate

**DOI:** 10.17533/udea.iee.v43n2e05

**Published:** 2025-07-23

**Authors:** Mohd. Parvez, Nipin Kalal, Dr. Mahendra Singh, Mrs. Khina Sharma

**Affiliations:** 1 RN, M.Sc. Email: parvezyakub00@gmail.com. https://orcid.org/0009-0008-3514-5353 parvezyakub00@gmail.com; 2 RN, M.Sc. Assistant professor. Email: kalalnipin@gmail.com. Corresponding author. https://orcid.org/0000-0002-1392-1787 All India Institute Of Medical Sciences India kalalnipin@gmail.com; 3 MD, DM. Associate Professor. Email: dr.mahi1118@gmail.com. https://orcid.org/0000-0001-8769-8269 All India Institute Of Medical Sciences India dr.mahi1118@gmail.com; 4 RN, M.Sc. Nursing Tutor. Email: khinasharma1043@gmail.com. https://orcid.org/0000-0003-3038-9742 All India Institute Of Medical Sciences India khinasharma1043@gmail.com; 5 College of nursing, AIIMS Jodhpur, Rajasthan, India. All India Institute Of Medical Sciences College of nursing AIIMS Jodhpur Rajasthan India; 6 Department of Urology AIIMS Jodhpur, Rajasthan, India. All India Institute Of Medical Sciences Department of Urology AIIMS Jodhpur Rajasthan India

**Keywords:** nurses, transurethral resection of prostate, non-randomized controlled trials as topic, control groups., enfermeras y enfermeros, resección transuretral de la próstata, ensayos clínicos controlados no aleatorios como asunto, grupos control., enfermeiras e enfermeiros, ressecção transuretral da próstata, ensaios clínicos controlados não aleatórios como assunto, grupos controle.

## Abstract

**Objective.:**

To assess the effectiveness of an educational intervention in reducing post-operative complications and improve functional outcome patients undergoing Transurethral Resection of the Prostate -TURP-.

**Methods.:**

A quasi-experimental nursing-led study (post-test only control design) was conducted among 60 TURP patients (intervention group *n*=30 and control group *n*=30). Face-to-face education on self-management strategies lasting 20-25 minutes, educational booklet and weekly telephone follow-up was administered to the intervention group; while control group received routine care. Data collection tool administered at 4th and 8th week post TURP included a post-operative checklist, IPSS (International Prostate Symptom Score), and IIEF (international index of erectile function).

**Results.:**

Urinary incontinence rates were considerably reduced in the experimental group at 4 weeks (*p<*0.001). Despite an 8-week reduction in differences, both groups Indicated effective care and no cases of trans-urethral resection syndrome or urinary tract infection. In the experimental group, IPSS scores were lower (9.9±4.6) than in the control group (15.6±5.8) indicating improved symptoms (*p*<0.001). Additionally, IIEF scores were higher in the experimental Group (3.5±1.2) than in the control group (2.6±1.2), suggesting improved erectile function with nurse-led intervention (*p*<0.004).

**Conclusion.:**

The nurse-led educational intervention effectively improves functional outcomes and reduce post-operative complications in Benign prostatic hyperplasia patients following TURP. Integrating such interventions by healthcare professionals can further accelerate recovery and minimize complications.

## Resumen

**Objetivo.:**

Evaluar la efectividad de una intervención educativa para reducir las complicaciones postoperatorias y mejorar los resultados funcionales de los pacientes sometidos a Resección Transuretral de Próstata -RTUP-.

**Métodos.:**

Se realizó un estudio cuasi-experimental liderado por enfermería, con grupo control solo en el post-test. Participaron 60 pacientes sometidos a RTUP (grupo intervención *n*=30 y grupo control *n*=30) en Rajasthan, India. Al grupo de intervención se le impartió educación por enfermería sobre estrategias de automanejo de 20-25 minutos de duración, un folleto educativo y seguimiento telefónico semanal; mientras que el grupo de control recibió atención rutinaria. La herramienta de recogida de datos fue administrada a la 4ª y 8ª semana tras la RTUP incluía una lista de comprobación postoperatoria IPSS (puntuación internacional de síntomas prostáticos) e IIFE (índice internacional de función eréctil).

**Resultados.:**

Las tasas de incontinencia urinaria se redujeron considerablemente en el grupo experimental a las 4 semanas (*p*<0.001). A pesar de que las diferencias se redujeron a las 8 semanas, ambos grupos indicaron una atención eficaz y ningún caso de síndrome de resección transuretral o infección urinaria. En el grupo experimental, las puntuaciones del IPSS fueron más bajas (9.9±4.6) que en el grupo de control (15.6±5.8), lo que indica una mejoría de los síntomas (*p*<0.001). Además, las puntuaciones del IIEF fueron mayores en el grupo experimental (3.5±1.2) que en el grupo de control (2.6±1.18), lo que sugiere una mejora de la función eréctil con la intervencin (*p*<0.004).

**Conclusiones.:**

La intervención educativa dirigida por enfermeras mejora eficazmente los resultados funcionales y reduce las complicaciones postoperatorias en pacientes con hiperplasia benigna de próstata tras una RTUP. La integración de este tipo de intervenciones por parte de los profesionales sanitarios puede acelerar aún más la recuperación y minimizar las complicaciones.

## Resumo

**Objetivo.:**

Avaliar a eficácia de uma intervenção educacional na redução de complicações pós-operatórias e na melhora dos resultados funcionais em pacientes submetidos à Ressecção Transuretral da Próstata (RTU).

**Métodos.:**

Um estudo quase experimental (com um grupo controle apenas no pós-teste) foi conduzido por enfermeiros com a participação de 60 pacientes submetidos à RTU (grupo intervenção *n*= 30 e grupo controle *n*= 30). O grupo intervenção recebeu educação presencial sobre estratégias de autogestão com duração de 20 a 25 minutos, um folheto educativo e acompanhamento semanal por telefone; enquanto o grupo controle recebeu cuidados de rotina. A ferramenta de coleta de dados administrada em 4 e 8 semanas após a RTU incluiu uma lista de verificação pós-operatória, IPSS (*International Prostate Symptom Score*) e IIEF (*International Index of Erectile Function*).

**Resultados.:**

As taxas de incontinência urinária foram significativamente reduzidas no grupo experimental em 4 semanas (*p*< 0.001). Embora as diferenças tenham diminuído em 8 semanas, ambos os grupos relataram cuidados eficazes e nenhum caso de síndrome de ressecção transuretral ou infecção do trato urinário. As pontuações do IPSS foram menores no grupo experimental (9.9 ± 4,6) do que no grupo controle (15.6±5,8), indicando melhora nos sintomas (*p*<0.001). Além disso, as pontuações do IIEF foram maiores no grupo experimental (3.5±1.2) do que no grupo controle (2.6±1.18), sugerindo melhora da função erétil com a intervenção liderada por enfermeiros (*p*<0.004).

**Conclusões.:**

A intervenção educacional liderada por enfermeiros melhora efetivamente os resultados funcionais e reduz as complicações pós-operatórias em pacientes com hiperplasia prostática benigna após TURP. A integração dessas intervenções por profissionais de saúde pode acelerar ainda mais a recuperação e minimizar as complicações.

## Introduction

Benign prostatic hyperplasia (BPH) is a common, noncancerous enlargement of the prostate gland that affects aging men, leading to urinary symptoms such as weak stream, urgency, frequency, hesitancy, and incontinence.^1^^,^^2^ Its prevalence increases with age, affecting approximately 15% of men over 40 and up to 60% of men by the age of 90.^1^ Treatment options include lifestyle modifications, pharmacological management, and surgical interventions, with transurethral resection of the prostate (TURP) being the gold standard for symptomatic relief.^2^^-^^5^While TURP is highly effective in improving urodynamic outcomes, it is associated with various complications, including early iatrogenic stress incontinence (30%-40%), urethral strictures (2.2%-9.8%), urinary retention, and erectile dysfunction (3.4%-32%).^3^^,^^6^. Additionally, TURP may lead to ejaculatory dysfunction (53%-75%) and psychological distress related to sexual health^5^^,^^7^^,^^8^ Postoperative complications such as urinary tract infections, bleeding, catheter blockage, bladder discomfort, and TUR syndrome due to fluid overload further impact recovery and quality of life.^1^^,^^5^^,^^6^ Given these challenges, comprehensive post-operative care is crucial to minimize complications and improve functional outcomes. 

Nurse-led interventions, including structured patient education on self-care, activity modification, nutrition, hygiene, and symptom management, play a vital role in enhancing recovery and overall well-being.^1^^,^^10^^,^^11^ However, limited research has been conducted in our country on the effectiveness of nursing education in reducing post-operative complications and optimizing functional outcomes following TURP.^9^^,^^10^^,^^12^This study aims to assess the impact of a nurse-led educational intervention on preventing post-operative complications and improving functional outcomes among patients undergoing TURP at a tertiary care centre. By addressing this research gap, the study seeks to highlight the essential role of nursing education in improving patient outcomes and advancing evidence-based nursing practices in surgical care.

## Methods

Study design. A quasi-experimental post-test control group study with experimental and control group was conducted from October 2023 to march 2024. The study was carried out in All India Institute of Medical Sciences, Jodhpur, India, and a sample size of 60 patients which accounted for a 10% dropout rate was established statistically^2^.The consecutive sampling technique was used for selection of participant.

Setting and Participants. The criteria for inclusion used to find participants: older than 40 year, able to understand Hindi or English, and willing to provide consent. Patients who were sexually inactive, had failed TURP, or were unable of understanding instructions were among the exclusion criteria.

Measures. Data were collected using a demographic with clinical variable sheet, international prostate symptom score, international index of erectile function questionnaire and self-develop post-operative checklist. (i) The International Prostate Symptom Score (IPSS), a validated tool with eight questions, evaluates BPH symptoms experienced in the past month such as incomplete bladder emptying and frequent urination. Scores ranging from 0 to 35 categorize symptom severity into mild (0-7), moderate (8-19), and severe (20-35); (ii) The International Index of Erectile Function (IIEF) questionnaire, a validated tool with 15 questions, is commonly used in clinical trials to evaluate treatment effects on erectile dysfunction. Each question, rated from 0 to 5, assesses four key domains of male sexual function: erectile function, orgasmic function, sexual desire, and intercourse satisfaction. Scores falling within specific ranges categorize patients' severity levels: severe (6-10), moderate (11-16), mild-to-moderate (17-21), mild (22-25) and no dysfunction (26-30); (iii)The self-developed post-operative checklist to asses UTI, urinary incontinent, hematuria, fever and TUR syndrome. This checklist gives us the information about how many patients underwent post-operative complications after TURP and checklist is validated by various medical experts. 

Validity and reliability. The tool, health education pamphlet underwent validation by medical and nursing experts. Furthermore, the tools were validated for Hindi by a Hindi literature expert. For reliability, the IPSS and the IIEF questionnaire were pre-validated, demonstrating Cronbach’s α values of 0.91^13^ and 0.828,^14^ respectively.

**Figure1:**
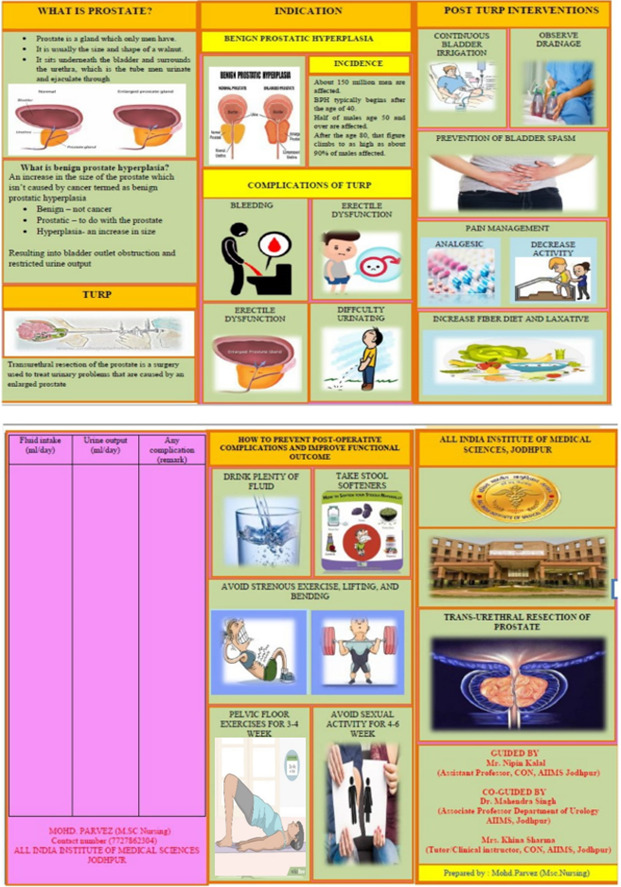
Prototype and content of the educational Pamphlet

Intervention. Control group participants received routine care for undergoing TURP as per the protocols of the urology IPD. This included discharge teaching by a senior resident related to post-operative complications. Experimental group participants received the following nurse-led intervention in addition to routine care for undergoing TURP: (i) Health Education: The Experimental group received health education on self-management strategies with help of PPT and face to face interview at 20-25 minute. These included recommendations to avoid strenuous activities, such as heavy lifting, for four to six weeks, refraining from moving heavy objects, abstaining from sexual activity for the last four to six weeks, and maintaining adequate hydration. Additionally, participants were advised on specific behavioural changes, such as bladder retraining (including pelvic floor exercises), double voiding, and urethral milking. Dietary changes were also recommended to prevent constipation; (ii) Pamphlet distribution: An educational pamphlet was provided to the experimental group patients immediately after the health education session. The pamphlet included the following components: definition of the procedure, benefits of the procedure, possible post-operative complications, all self-management strategies and specific changes in behavior explained above, and expected future complications. (Figure1); (iii) Telephonic follow-up: Telephonic confirmation and reinforcement were done once weekly to ensure the compliance of interventions taught to the patients during the nurse-led educational intervention.

Post-intervention data collection. At 4 week’s post-intervention, post-operative complications were assessed in both the control and experimental groups. The IPS questionnaire was also administered to both groups. At 8 weeks post-intervention, post-operative complications were again assessed in both the control and experimental groups and the IIEF questionnaire. Both sets of data were collected through direct face-to-face interviews, utilizing a combination of self-report and interview methods.

Data analysis. Descriptive statistics (frequency, percentage, mean, and standard deviation) and inferential statistics (independent simple t-tests, chi-square and Fisher's exact test) were used to test hypotheses and compare groups during data analysis using SPSS v20. P value less than 0.05 was considered statistically significant.

## Results

Table 1 presents the sociodemographic and clinical characteristics associated with the individuals who were part of the experimental and control groups. The distributions of all were comparable in both groups, suggesting homogeneity. Most participants were between 61-80 years in both groups; control (73.3%) and experimental (60%) (*p*=0.71). 80% of the control and 70% of the experimental group were self-employed (*p*=0.28) and 66.7% from rural areas in both groups (*p*=0.45). 46.7% of the control group and 56.7% of the experimental group had no comorbidities and above one third in both the groups were hypertensive (*p*=0.11). Previous urethral surgery was reported in 13.3% of control and 20% of experimental group (*p* = 0.11). 63.3% of the control group and 73.3% of the experimental group (*p*=0.95) reported a normal PSA level (<4 ng/ml). Uroflowmetry results were <10ml/sec in 73.3% of control and 56.7% of experimental group participants (*p*=0.26). These results imply that the study's participant pool was well-matched, ensuring uniformity in the assessment of therapeutic results and enabling reliable comparisons between the experimental and control groups. 

**Table 1:** Sociodemographic and clinical variables of the participants by group

Variables	Control group (*n*=30)	**Experimental group (*n*=30)**	Total (*n*=60)	***p*-value** **(chi-square)**
Sociodemographic variables	f (%)	f (%)	f (%)	
Age (Years) 40-60 61-80	8 (26.7) 22 (73.3)	12 (40) 18 (60)	20 (33.3) 40 (66.7)	0.71
Education No formal education Primary Secondary and senior secondary Graduation	15 (50) 2 (6.7) 7 (23.3) 6 (20)	13 (43.3) 10 (33.3) 4 (13.3) 3 (10.1)	28 (46.7) 12 (20.0) 11 (18.3) 9 (15.0)	0.97
Occupation Unemployed Self- employed Govt. service and other	2 (6.7) 24 (80.0) 4 (13.3)	5 (16.7) 21 (70.0) 4 (13.3)	7 (11.7) 45 (75.0) 8 (13.3)	0.28
Area of residence Urban Rural	10 (33.3) 20 (66.7)	10 (33.3) 20 (66.7)	20 (33.3) 40 (66.7)	0.45
Clinical variables				
Duration of symptom < 6 months > 6 months	9 (30.0) 21 (70.0)	8 (26.7) 22 (73.3)	17 (28.3) 43 (71.7)	0.72
Bowel habits (constipation) No Yes	12 (40.0) 18 (60.0)	9 (30.0) 21 (70.0)	21 (35.0) 39 (65.0)	0.71
Any comorbidities No Hypertension Diabetes Mellitus	14 (46.7) 10 (33.3) 6 (20.0)	17 (56.7) 9 (30.0) 4 (13.3)	31 (51.7) 19 (31.7) 10 (16.6)	0.11
Previous intervention (per urethral surgery) Yes No	4 (13.3) 26 (86.7)	6 (20.0) 24 (80.0)	10 (16.6) 50 (83.3)	0.11
Prostate specific antigen (ng/ml) <4 4-10	19 (63.3) 11 (36.7)	22 (73.3) 8 (26.7)	41 (68.3) 19 (31.7)	0.95
USG-KUB (prostate volume) <20cc 20-40cc >40cc	4 (13.3) 13 (43.3) 13 (43.3)	5 (16.7) 10 (33.3) 15 (50.0)	9 (15.0) 23 (38.3) 28 (46.7)	0.44
Uroflowmetry (Qmax.ml/sec) >15 10-15 <10	3 (10.0) 5 (16.7) 22 (73.3)	2 (6.7) 11 (36.7) 17 (56.7)	5 (8.3) 16 (26.7) 39 (65.0)	0.26
BPH (grade) 1 2 3	5 (16.7) 16 (53.3) 9 (30.0)	9 (30.0) 15 (50.0) 6 (20.0)	14 (23.4) 31 (51.6) 15 (25.0)	0.14

USG-KUB: Ultrasound of the kidney, ureters and bladder

Table 2 shows that After four weeks; the experimental group exceeded the control group in terms of both quality of life and reduced intensity of symptoms. The experimental group exhibited a notable decrease with an average IPSS score of 9.9 ± 4.6, which was considerably lower than the control group's score of 15.6 ± 5.8 (*p*<0.001). In addition, the IPSS-QOL index mean for the experimental group was 1.6 ± 0.9, significantly higher than the control group's 2.7 ± 0.9 (*p*<0.001), indicating a much higher quality of life. These results highlight the experimental intervention's clinical value in improving participants' overall well-being and reducing symptoms of post-operative complications of TURP. The experimental group's erectile function significantly improved when compared to the control group. At 8 weeks the control groups mean International Index of Erectile Function (IIEF) score was 2.6 ± 1.18, while the experimental group's score was higher at 3.5 ± 1.2 (*p*<0.004). This demonstrates how well the nurse-led intervention improved erectile function. Post-operative complications after TURP are presented in Table 3 for both the experimental and control groups. Urinary incontinence rates were considerably reduced in the experimental group at 4 weeks (*p*=0.001). Despite an 8-week reduction in differences, both groups Indicated effective care and no cases of TUR (trans-urethral resection syndrome) or UTI (urinary tract infection)

**Table 2:** Comparison the intervention effects on the International Prostate Symptom Score (IPSS) and International index of erectile function (IIEF) between the Control and Experimental groups.

Variables	Control group (*n*=30) Mean ±SD	Experimental group (*n*=30) Mean ±SD	*p*-value*
IPSS score - After 4 weeks	15.6±5.8	9.9±4.6	<0.001
IPSS-QOL index - After 4 weeks	2.7±0.9	1.6±0.9	<0.001
IIEF score - After 8 weeks	2.6±1.18	3.5±1.2	<0.004

(*) Independent sample t-test; IPSS (Min-Max score = 0 to 35); IPSS-QOL Index (Min-Max score = 0-6)

**Table 3:** Comparison of post-operative complications among Control and Experimental groups after 4 and 8 weeks of TURP

Variables	Control group (n=30) f (%)	Experimental group N(30) f (%)	***p*-value** **( chi-square)**	Control group (n=30) f (%)	Experimental group (n=30) f (%)	***p*-value** **(chi-square)**
	After 4 weeks of TURP			After 8 weeks of TURP		
Hematuria Yes No	8 (26.7) 22 (73.3)	3 (10.0) 27 (90.0)	0.09	10 (33.3) 20 (66.7)	7 (23.3) 23 (76.7)	0.39
Urinary incontinace Yes No	13 (43.3) 17 (56.7)	2 (6.7) 28 (93.3)	0.001	19 (63.3) 1 1(36.7)	16 (53.3) 14 (46.7)	0.43
Fever Yes No	4 (13.3) 26 (86.7)	1 (3.3) 29 (96.7)	0.35	7 (23.3) 23 (76.7)	6 (20.0) 24 (80.0)	0.75

## Discussion

The present study aimed to evaluate the impact of a nurse-led educational intervention on post-operative outcomes in patients undergoing Transurethral Resection of the Prostate (TURP). Demographic characteristics were comparable across the control and experimental groups. Most participants were aged 61-80 and had been experiencing symptoms of benign prostatic hyperplasia (BPH) for over six months. Similar findings have been reported by Khalil *et al.*^1^ and Begla *et al.,*^4^, though the age range in their studies tended to be slightly younger. Prostate-specific antigen (PSA) levels were normal in over two-thirds of participants, aligning with Chaudhary *et al.,*^2^ who found average PSA levels of 2.6 ng/ml and 1.4 ng/ml in their cohorts. In our study, 46.7% of participants had prostate volumes exceeding 40 cc, which contrasts with the 20-40 cc average noted in Chaudhary *et al.,*^2^ suggesting that prostate volume alone may not directly correlate with symptom severity. Additionally, our study found that most patients (65%) had a low maximum flow rate of urine (<10ml/sec), similar to Chaudhary *et al*.^2^ reinforcing that BPH is often associated with decreased urinary flow and the subsequent need for TURP.

The study examined the occurrence of post-operative complications following TURP procedure, including haematuria (blood in urine), UTIs, urinary incontinence, fever, and TUR syndrome. In our study, we found no significant difference in post-operative TURP complications, except for the persistence of urinary incontinence after 4 weeks of intervention. Our findings indicated that haematuria was less frequent after four weeks in both the control and experimental groups compared to findings in a study by Khali *et al*.^1^ Similarly, urinary incontinence was observed in the control group after four weeks; our findings contrasted with the result by Khali *et al*.^1^ where it was not reported in either group. We also found that a nurse-led educational intervention effectively prevented fever and urinary incontinence incidence in the experimental group compared to the control group. Notably, we did not observe any UTIs or cases of TUR syndrome during the study period. The risk of UTIs could be minimized through prolonged use of prophylactic antibiotics, while advanced procedures and maintenance of electrolyte balance during the intervention could help reduce the risk of TUR Syndrome. In contrast to our findings, Rassweiler *et al*.^15^ reported that certain complications, such as UTIs and haematuria, might occur after six weeks following TURP.

The mean IPSS score decreased in the experimental group (9.9 ± 4.6), suggesting a beneficial effect of the educational intervention. These findings are in line with Chaudhary *et al.,*^2^ who also observed a significant reduction in the IPSS following a nurse-led intervention. The IPSS-QOL index and IIEF scores showed statistically significant improvements in the experimental group, further indicating the intervention’s efficacy in improving quality of life and functional outcomes. Similar results were observed by Bayat *et al*.,^3^ who emphasized the benefits of pelvic floor exercises as part of post-operative care. The study findings suggest that a structured, nurse-led educational intervention positively affects both functional outcomes and post-operative complications. Moreover, the outcomes of our study align with those reported by Bayat *et al.*^3^ However, no previous study has specifically examined the impact of nurse-led educational interventions on both post-operative complications and functional outcomes in patients undergoing TURP. This study demonstrates that such interventions can effectively improve functional outcomes and reduce post-operative complications in TURP patients.

Conclusion. In recent years, transurethral prostate resection (TURER) has been the gold standard treatment for benign prostatic hyperplasia (BPH). However, there have been significant changes in self-care practices, mainly due to the emergence of serious post-operative complications. Engaging in self-care and adopting a healthy lifestyle not only helps prevent post-operative complications, but also improves functional outcomes. 

The results of this study underscore the benefits of a nurse-led educational intervention. However, it was found that concurrent nurse-led education further improved functional outcomes and reduced the incidence of postoperative complications. It is therefore strongly recommended that comprehensive health education is provided by health professionals. Such an approach may lead to more substantial improvements in a shorter period of time, reducing the severity of complications and improving functional outcomes for patients following TURP.
